# *Boswellia carterii* n-hexane extract suppresses breast cancer growth via induction of ferroptosis by downregulated GPX4 and upregulated transferrin

**DOI:** 10.1038/s41598-024-65170-6

**Published:** 2024-06-21

**Authors:** Jinxin Xie, Huiming Huang, Xuejiao Wei, Peng Tan, Lishan Ouyang, Longyan Wang, Dongxiao Liu, Fei Wang, Zhuguo Wang, Pengfei Tu, Jun Li, Xiaojun Zha, Zhongdong Hu

**Affiliations:** 1 School of Chinese Materia Medica, Beijing University of Chinese Medicine, Beijing, 100029 China; 2 Modern Research Center for Traditional Chinese Medicine, Beijing Research Institute of Chinese Medicine, Beijing University of Chinese Medicine, Beijing, 100029 China; 3 Department of Biochemistry & Molecular Biology, School of Basic Medicine, Anhui Medical University, Hefei, 230032 China

**Keywords:** *Boswellia carterii* n-hexane extract, Breast cancer, Ferroptosis, GPX4, Transferrin, Breast cancer, Cancer therapy, Drug development

## Abstract

Breast cancer (BC) remains a significant health concern for women globally, prompting the relentless pursuit of novel therapeutic modalities. As a traditional Chinese medicine, *Boswellia carterii* has been extensively used to treat various cancers, such as BC. However, the anti-BC effect and underlying mechanism of *Boswellia carterii* remain largely unclear. The aim of this study is to explore the therapeutic effect of *Boswellia carterii* n-hexane extract (BCHE) against BC as well as its underlying mechanism. The present study showed that BCHE significantly suppressed the viability of human BC cells. Moreover, BCHE exhibited potent anti-BC activity in vivo with no significant toxic effects. Additionally, BCHE induced ferroptosis via increased Transferrin expression and the intracellular accumulation of Fe^2+^, as well as decreased glutathione peroxidase 4 (GPX4) expression and the upregulation of reactive oxygen species (ROS)-induced lipid peroxidation in BC cells. In vivo experimental results also demonstrated that BCHE effectively induced ferroptosis through GPX4 downregulation and Transferrin upregulation in tumor-bearing mice. Overall, BCHE inhibited the growth of BC cells by inducing ferroptosis mediated by modulating the iron accumulation pathway and the lipid peroxidation pathway. Therefore, BCHE could serve as a potential ferroptosis-targeting drug for treating BC.

## Introduction

Breast cancer (BC) is a prominent cause of death in females worldwide. In 2020, BC had the greatest incidence of various cancer types in women. New cases of BC and deaths due to BC constitute approximately 12.5% and 6.92% of all cases, respectively^1^. Among the newly diagnosed cases of BC, the proportion of cases with initially diagnosed advanced-stage cancer ranges from 3 to 10%. However, in early-stage BC, there is still a 30–40% risk of metastasis or recurrence even after the patient undergoes systematic standardized treatment^2^. Consequently, there is an urgent need for novel therapeutic drugs to treat BC.

*Boswellia carterii* is obtained from the dried resin exuded from the tree bark of *Boswellia carterii* Birdw., which belongs to the *Burseraceae* family. In China, *Boswellia carterii* is extensively used to treat various cancers, such as BC^3^. The Xihuang pill, which includes *Boswellia carterii* as one of its main components, is a commonly used anticancer medication in clinical practice and is used for treating different cancers, such as BC, lung cancer, and nasopharyngeal carcinoma, in China^4^. *Boswellia carterii* shows antitumor activity by arresting cell cycle progression and inhibiting tumor cell proliferation, angiogenesis, invasion, and migration^5^. *Boswellia carterii* essential oil hinders cell growth while inducing human BC cell apoptosis through the AMPK/mTOR pathway^6^. However, in-depth research on the anti-BC effects of *Boswellia carterii* is lacking. Additionally, the active ingredients of *Boswellia carterii* responsible for its anticancer effects and the specific underlying mechanisms of action remain unclear.

Ferroptosis is an iron-dependent form of cell death that involves excessive iron accumulation. Lipid peroxidation and increased reactive oxygen species (ROS) production occur during ferroptosis^7^. There is evidence indicating that targeting ferroptosis may become a potentially effective treatment strategy for BC^7,8^.

In this work, *Boswellia carterii* n-hexane extract (BCHE) exhibited potent anti-BC efficacy both in vitro and in vivo. Moreover, BCHE promoted ferroptosis in BC. BCHE upregulated Transferrin expression, promoted iron accumulation, and increased ROS levels, thereby leading to lipid peroxidation in human BC cells. In addition, decreased glutathione peroxidase (GPX4) expression was involved in the induction of ferroptosis by BCHE. Taken together, BCHE inhibited the growth of BC cells by inducing ferroptosis mediated by GPX4 downregulation and Transferrin upregulation.

## Materials and methods

### Reagents and antibodies

RPMI-1640 (C11875500BT) and TrypLE™ Express (12604021) were obtained from Gibco (New York, USA). DMEM (10-013-CV) and FBS (35-081-cv) were purchased from Corning (New York, USA). 3-Methyladenine (HY-19312), necrostatin-1 (HY-15760), Z-VAD-FMK (HY-16658B), ferrostatin-1 (HY-100579), and deferoxamine (HY-B1625) were obtained from MCE (New Jersey, USA). ECL chemiluminescent solution (BN30793 and BN16010) was obtained from Biorigin (Beijing, China). PVDF membranes were purchased from Millipore (Massachusetts, USA). The following primary antibodies were used: anti-β-actin and anti-GPX4 from Santa Cruz Biologicals (California, USA), anti-transferrin from Hangzhou Jingjie Biotechnology Co., Ltd. (Hangzhou, China), and anti-GAPDH from Abcam (Cambridge, United Kingdom). HRP-conjugated goat anti-mouse/rabbit secondary antibodies were obtained from Santa Cruz Biologicals (California, USA).

### Cell culture

Human BC cells (MDA-MB-231 and MCF-7) were obtained from the Cell Center, Institute of Basic Medical Sciences, Chinese Academy of Medical Sciences. The 4T1 cells and human normal breast epithelial cells (MCF-10A) were obtained from the American Type Culture Collection (Manassas, ATCC). The cells were cultivated in RPMI-1640 and DMEM supplemented with 10% fetal bovine serum, while MCF-10A cells were specifically nurtured in a customized medium that was enriched with 20 ng/mL EGF, Hydrocortisone, Insulin, NEAA, and 5% HS. All cultures were maintained under sterile conditions at 37 °C with 5% CO_2_.

### Preparation of BCHE

*Boswellia carterii* (No. 94380702) was purchased from Beijing Tong Ren Tang and subjected to sequential extraction by refluxing with n-hexane, dichloromethane, and ethanol for 2 h, followed by rotary evaporation of the solvent. The obtained extract was then dissolved in DMSO to prepare a 50 mg/mL master batch and kept aside. The composition of BCHE was identified by GC–MS, and the constituents of BCHE mainly included diterpenoids, pentacyclic triterpenoids and steroids (Supplementary Figure S1). In previous studies, 49 compounds were separated from *Boswellia carterii* hexane extract, including diterpenes, tetracyclic triterpenes, and pentacyclic triterpenes. Using GC–MS and referencing 16 compounds, a total of 28 compounds were identified, primarily consisting of active ingredients such as octyl acetate, dodecanoic acid, tetradecanoic acid, cembrene, isocembrol, and cholesta-2,4-diene^9–11^.

### Measurement of MTT

After the digestion and dilution of the densely grown MDA-MB-231 and MCF-7 cells, the cells were counted using a cell counter, diluted to 3 × 10^4^ cells/mL and subsequently incubated in a 96-well plate for 24 h. The complete medium was then used to dilute the stock solution of BCHE (50 mg/mL) to obtain working solutions with various concentrations for drug treatment. At 24, 48, and 72 h after drug administration, cell viability was determined with an MTT assay kit (0793, LABLEAD, Beijing, China) in accordance with specific protocols.

### Measurement of colony formation

The cells were digested and counted using the abovementioned method. A total of 1000 cells were inoculated into each 6-cm dish. At 24 h of post-adherence, the culture medium was replaced with or without BCHE. Three replicate dishes were used for each group, and cell culture was performed in the respective drug-containing media every 3–4 days. After drug administration for 12 days, the cells were rinsed three times with PBS. Following fixation using a cell tissue fixative (BN20094, Biorigin, Beijing, China), crystal violet (G1063, Solarbio, Beijing, China) was added for cell staining. The stained cells were then imaged and counted.

### Measurement of EdU

Cells at an appropriate density were inoculated into a 6-well plate. Following 24 h of adherence, medium containing varying doses of BCHE was added to each well. After 48 h, the cells were processed with the BeyoClick™ EdU Cell Proliferation Kit using Alexa Fluor 488 (C0071S, Beyotime Biotechnology, Shanghai, China) in accordance with specific protocols. The 6-well plate was then observed under a fluorescence inverted microscope to assess how BCHE affects human BC cell proliferation.

### Measurement of ROS

After cell counting, the cells at an appropriate concentration were inoculated into a 6-well plate. After overnight adherence, the cells were subjected to 48 h of drug administration. Subsequently, after trypsin digestion, the intracellular ROS levels were measured with the ROS detection kit (D6470, Solarbio, Beijing, China) according to the manufacturer’s instructions. Using the ROS-sensitive fluorescent probe DCFH-DA, the ROS levels were measured with a flow cytometer (FACS Canto II, BD Biosciences, New Jersey, USA).

### Fe^2+^ level measurement

The cells at an appropriate density were inoculated into a cell culture plate. Following overnight adherence, the cells were subjected to 48 h of drug stimulation. We subsequently measured changes in intracellular Fe^2+^ levels before and after BCHE treatment with a Cell Ferrous Iron Colorimetric Assay Kit (E-BC-K881-M, Elabscience, Wuhan, China) according to the manufacturer’s instructions. Furthermore, the changes in Fe^2+^ levels in animal tumor tissues after BCHE treatment were determined by a Ferrous Iron Colorimetric Assay Kit (E-BC-K773-M, Elabscience, Wuhan, China). The relative content of Fe^2+^ was detected using the Fe^2+^-specific fluorescent probe FerroOrange (F374, Dojindo Laboratories, Kanagawa, Japan) with a flow cytometer (LSRFortessa™, BD Biosciences, New Jersey, USA) and qualitatively monitored with a laser confocal microscope (Leica TCS SP8 X, Leica, Wetzlar, Germany) (10 × objective lens, 40 × magnification, 2 × zoom).

### Measurement of lipid peroxidation

The abovementioned procedure was followed for cell seeding and drug treatment. After the completion of drug treatment, changes in the lipid peroxide levels in the cells were measured using the lipid peroxidation detection probe BDP 581/591 C11 (L267, Dojindo Laboratories, Kanagawa, Japan) in accordance with specific protocols. The measurement was performed with a flow cytometer, and the relative content of lipid peroxides in the cells following drug treatment was quantitatively assessed. Additionally, lipid peroxides were qualitatively assessed using a laser confocal microscope.

### Transcriptome sequencing analysis

Total RNA was extracted from MDA-MB-231 cells subjected to treatment with or without 40 μg/mL BCHE for 24 h with RNA-Solv Reagent (L01UM, Omega Biotek, Georgia, USA). Illumina HiSeq 2500 mRNA sequencing was conducted at Shanghai Biotechnology Corporation (Shanghai, China), and HISAT2 and Stringtie software were used for data analysis as described earlier^12^.

### Quantitative real-time PCR (qRT-PCR)

Total cellular RNA was isolated with/without 24 h BCHE treatment at specific concentrations. RNA extraction was performed using RNA-Solv Reagent (L01UM, Omega Biotek, Georgia, USA). Subsequently, the extracted RNA was used to prepare cDNA with the PrimeScript RT Reagent Kit according to the manufacturer’s protocol. This assay was carried out according to previous methods. Then, qRT‒PCR was carried out with primers obtained from Beijing Genomics Institute (Beijing, China), and the sequences of primers used were as follows:

GPX4 Forward: GAGGCAAGACCGAAGTAAACTAC;

GPX4 reverse: CCGAACTGGTTACACGGGAA.

Transferrin Forward: GTGTGCAGTGTCGGAGCAT;

Transferrin reverse: CATCGGATGGAATGACGCTTT.

GAPDH Forward: GGAGCGAGATCCCTCCAAAAT;

GAPDH reverse: GGCTGTTGTCATACTTCTCATGG.

### Western blotting

The abovementioned procedure was followed for cell seeding and drug treatment. Following the completion of drug stimulation, the cells were rinsed three times with PBS before being placed on ice. Cell lysis buffer was then used for cell lysis, followed by transfer to 1.5 mL Eppendorf tubes. After heating for a 10 min period at 98 °C, the tubes were cooled to ambient temperature. The lysates were stored in a -40 °C freezer. The tumor tissues were harvested, immediately immersed in liquid nitrogen, and placed in a − 80 °C freezer. The frozen tumor tissue was then minced under low-temperature conditions to extract proteins. The minced tissue was subsequently added to RIPA lysis buffer and homogenized using a tissue homogenizer (Schwingmühle Tissue Lyser II, Qiagen GmbH, Hilden, Germany). The homogenized mixture was subjected to centrifugation for 5 min at 1000 rpm at 4 °C by using a high-speed refrigerated centrifuge (Eppendorf AG 22331 Eppendorf, Hamburg, Germany). The supernatant was collected and quantified with a bicinchoninic acid (BCA) protein detection kit (bn27109, Biorigin, Beijing, China). On the basis of the quantification results, loading buffer and additional RIPA lysis buffer (bn25011, Biorigin, Beijing, China) were mixed with the sample. Protein samples were then subjected to heating for 10 min at 98 °C and preserved in a − 40 °C freezer. As described previously, a western blotting assay was performed to measure protein expression^13^.

### Evaluation of BCHE efficacy in a breast cancer orthotopic transplantation model

Four- to five-week-old female BALB/c mice were obtained from Spafas Biological Technology Co., Ltd. (Beijing, China), and orthotopically implanted with 1 × 10^6^/100 µL of mouse breast cancer 4T1 cells beneath the mammary fat pad. When the tumor size reached 100–200 mm^3^, the animals were randomized into four groups (n = 6 per group): (a) control group: PBS was administered orally (gavage) once daily; (b) BCHE low-dose group: 400 mg/kg of BCHE was administered orally (gavage) once daily; (c) BCHE high-dose group: 800 mg/kg of BCHE was administered orally (gavage) once daily; and (d) paclitaxel (ip0020, Solarbio, Beijing, China) positive control group: 10 mg/kg of paclitaxel was administered intraperitoneally twice weekly. Following the drug administration, the mouse growth status, body weight, and tumor volume were monitored daily. The tumor volume (V) was determined as V (mm^3^) = (length × width^2^)/2. Following drug administration, one portion of the tumor tissues and tissues from the major organs were dissected and immersed in 4% paraformaldehyde. The remaining tumor tissue was snap-frozen in liquid nitrogen before preservation at − 80 °C. The fixed tissues were subjected to hematoxylin and eosin (H&E) staining and immunohistochemistry (IHC), while freshly preserved tumor tissues were subjected to western blotting and Fe^2+^ detection assays. All animal experiments were carried out in accordance with the guidelines for animal use and care approved by the Ethics Committee of Beijing University of Chinese Medicine (Approval No. BUCM-4-2023010302-1004).

### H&E and IHC staining

Tumor tissues and major organ tissues from mice in the control and BCHE groups were fixed with 4% paraformaldehyde. After dehydration with a gradient series of ethanol and xylene, the samples were subjected to paraffin embedding and sectioning into 5-µm-thick sections for subsequent H&E and IHC staining. IHC staining was carried out according to an earlier description^14^.

### Statistical analysis

The results were explored using GraphPad Prism 8.0 statistical software and are presented as the means ± SDs. Student’s *t* test or one-way analysis of variance (ANOVA) with Dunnett's multiple comparisons test was used for comparing between-group differences. *p* < 0.05 indicated statistical significance.

## Results

### BCHE suppressed human breast cancer MCF-7 and MDA-MB-231 cell growth

**Figure 1:**
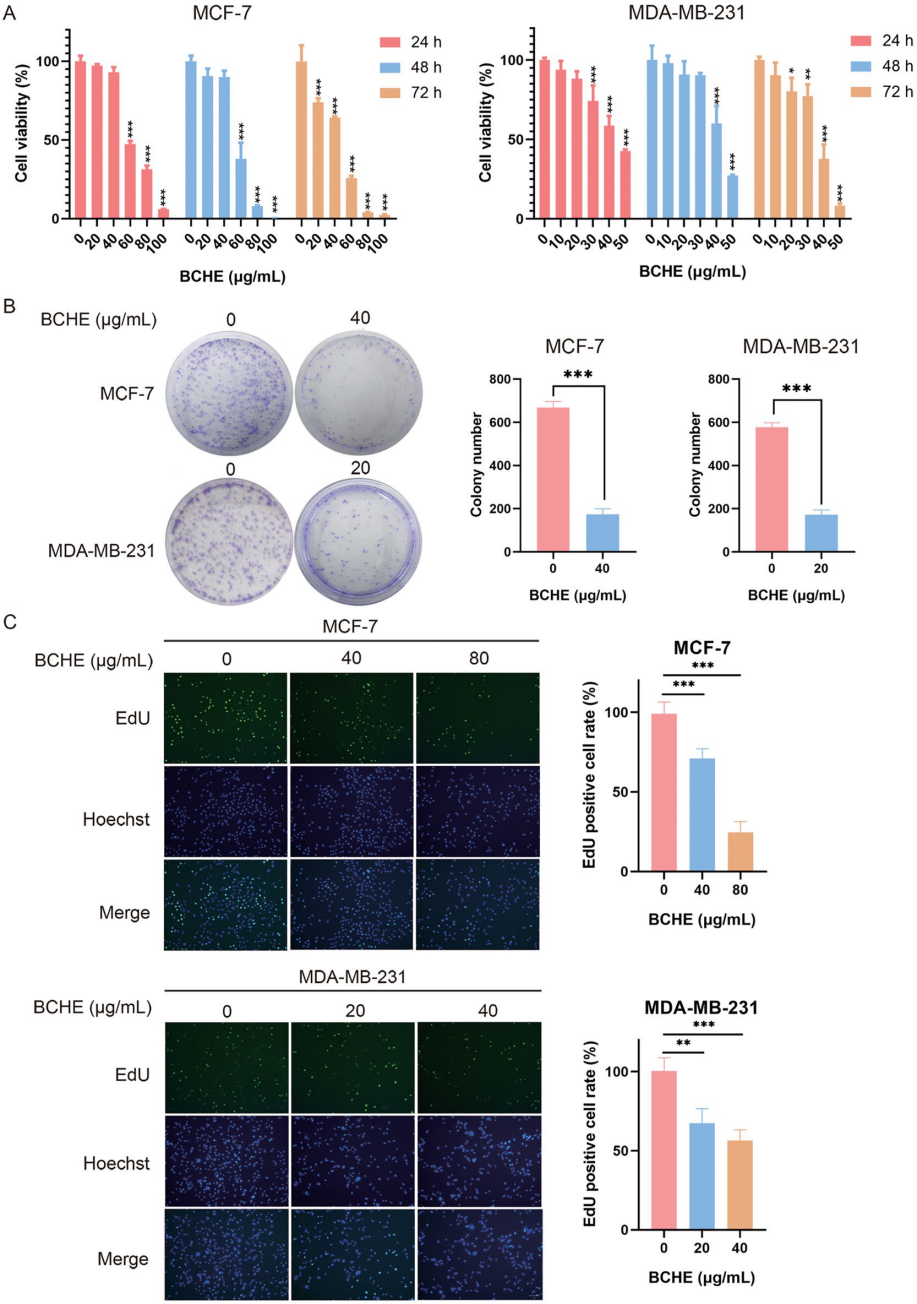
BCHE hindered human breast cancer MCF-7 and MDA-MB-231 cells proliferation. (**a**) BCHE was added to MCF-7 and MDA-MB-231 cells for 24, 48, and 72 h, and a MTT assay was then conducted to analyze cell viability. (**b**) These BC cells were subjected to BCHE (20 and 40 µg/mL) treatment for 12 days, followed by colony formation determination. The left and right panels show the typical imaging and quantitative findings, respectively. ^*^P < 0.05, ^**^P < 0.01, ^***^P < 0.001. (**c**) MCF-7 and MDA-MB-231 cells were exposed to 48 h of BCHE treatment, followed by EdU and Hoechst 33342 staining. EdU-labeled cells were detected as green fluorescence (488 nm), while the nuclei were stained with Hoechst 33342. The left and right panels show representative images (200 ×) and percentages of EdU-positive cells, respectively.

As revealed by the MTT assay, BCHE apparently suppressed human BC MDA-MB-231 and MCF-7 cell viability in a dose- and time-dependent manner (Fig. 1A), and the effect of BCHE on the cell viability of human normal breast epithelial MCF-10A cells was shown in Supplementary Figure S2. Additionally, BCHE markedly decreased the clonogenic ability of these cells (Fig. 1B). EdU staining was used to label proliferating cells with green fluorescence. According to our results, BCHE significantly hindered BC cell proliferation (Fig. 1C).

### Ferroptosis was implicated in BCHE-induced cell death in human BC cells

**Figure 2:**
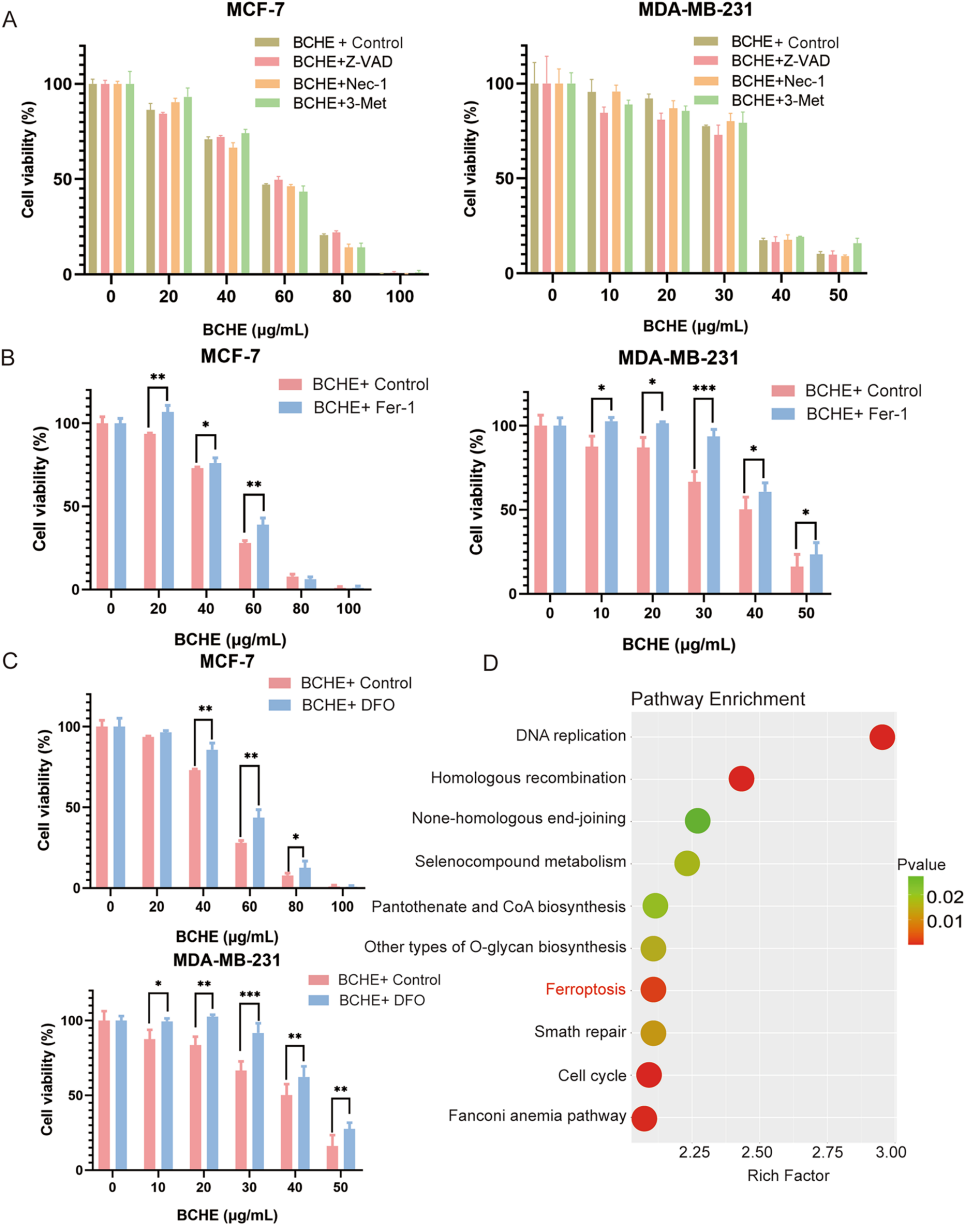
Ferroptosis was implicated in BCHE-induced death of human BC cells. (**a**) BCHE was added to treat MCF-7 and MDA-MB-231 cells for 48 h with/without 3-methyladenine (100 nM), necrostatin-1 (10 µM), and Z-VAD-FMK (10 µM). A MTT assay was conducted to explore cell viability. (**b**) Both BC cell lines were subjected to 48 h of BCHE treatment with or without ferrostatin-1 (10 µM). (**c**) Both BC cell lines were exposed to 48 h of BCHE treatment with or without deferoxamine (10 µM). (**d**) KEGG analysis of representative signaling pathway enrichment between different treatment groups of MDA-MB-231 cells. ^*^P < 0.05, ^**^P < 0.01, ^***^P < 0.001.

To investigate the mechanism related to BCHE-mediated cell death in BC cells, the above two BC cell lines were subjected to BCHE treatment with Z-VAD-FMK (an apoptosis inhibitor), necrostatin-1 (a necroptosis inhibitor), 3-methyladenine (an autophagy inhibitor), ferrostatin-1 (a ferroptosis inhibitor), or deferoxamine (a ferroptosis inhibitor). As depicted in Fig. 2A, BCHE-mediated cell death remained unaffected after treatment with Z-VAD-FMK, necrostatin-1, or 3-methyladenine in both BC cell lines. However, ferrostatin-1 (Fer-1) or deferoxamine (DFO) treatment attenuated the susceptibility of human BC cells to BCHE treatment (Fig. 2B and C). Moreover, RNA sequencing analysis revealed that ferroptosis may be regulated by BCHE in human BC cells (Fig. 2D). Overall, BCHE-induced cell death in human BC cells was partially mediated by ferroptosis.

### BCHE triggered ferroptosis by inducing intracellular iron and lipid peroxide accumulation in human BC cells

**Figure 3:**
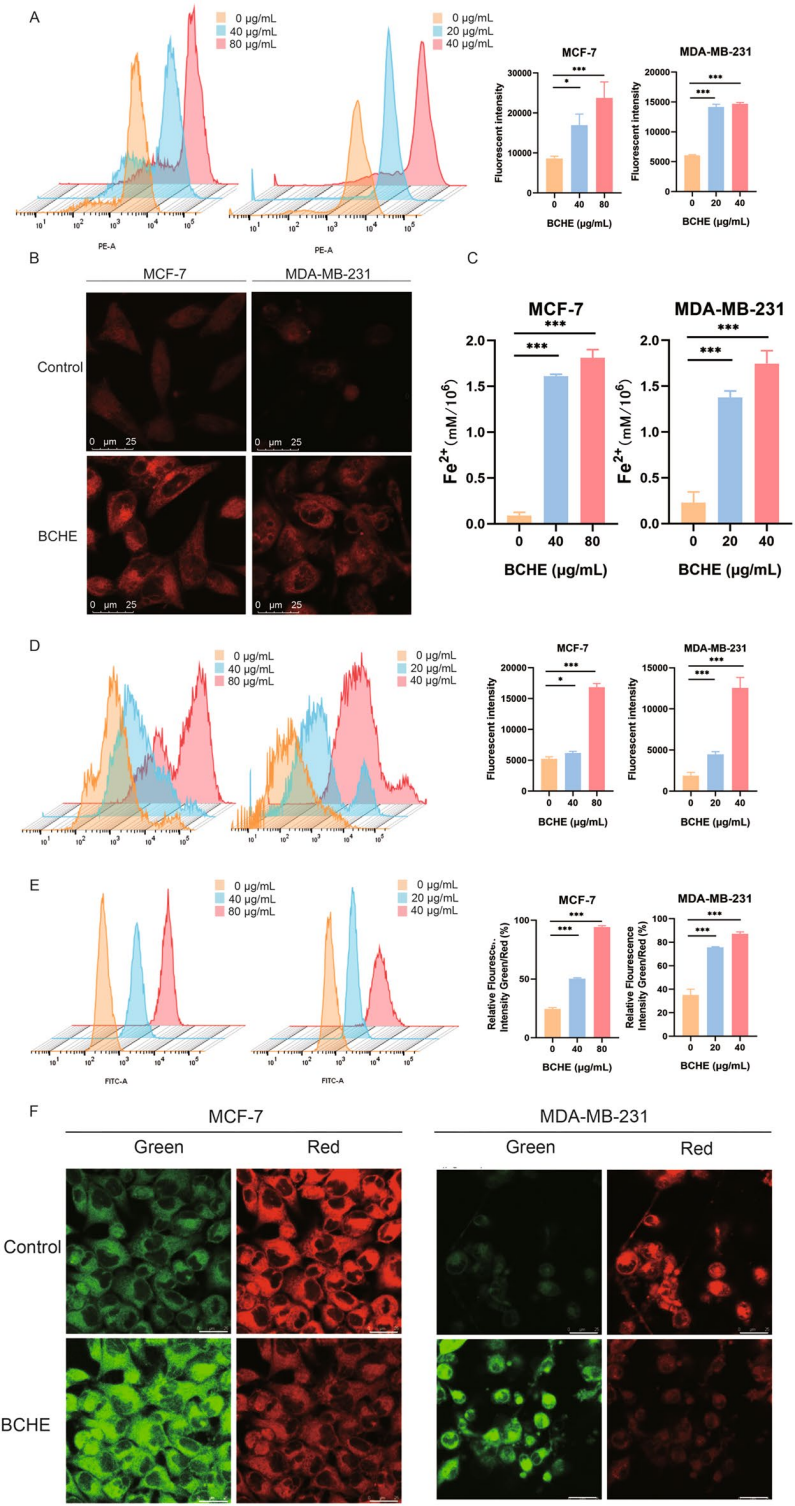
BCHE triggered ferroptosis by inducing intracellular iron and lipid peroxide accumulation in human BC cells. (**a**) Intracellular Fe^2+^ levels in BCHE-treated human BC cells were detected by flow cytometry. (**b**) Laser confocal microscopy was used to observe changes in intracellular Fe^2+^ levels in human BC cells after BCHE treatment (MCF-7, 40 µg/mL; MDA-MB-231, 20 µg/mL). The scale bar represents 25 µm. (**c**) A Fe^2+^ detection kit was used to measure the intracellular Fe^2+^ content in human BC cells. (**d**) Intracellular ROS content in BCHE-treated BC cells was detected via flow cytometry. (**e**) The levels of intracellular lipid peroxide in human BC cells following BCHE treatment were detected by flow cytometry. (**f**) Laser confocal microscopy was used to observe changes in intracellular lipid peroxide levels in human BC cells after BCHE treatment (MCF-7, 40 µg/mL; MDA-MB-231, 20 µg/mL). Green: FITC filter; Red: Texas Red filter; Scale bar represents 25 µm. ^*^P < 0.05, ^**^P < 0.01, ^***^P < 0.001.

Next, we focused on investigating the effects of BCHE on ferroptosis in human BC cells. Flow cytometry analysis revealed significantly elevated intracellular Fe^2+^ content in both BC cell lines following BCHE treatment (Fig. 3A). Additionally, intracellular Fe^2+^ levels were elevated in both cell lines following BCHE administration, as observed through laser confocal microscopy (Fig. 3B). To further validate this result, an Fe^2+^ detection kit was used to measure the intracellular Fe^2+^ content in BC cells treated with or without BCHE, and consistent results were obtained (Fig. 3C). Thus, BCHE significantly elevated the intracellular Fe^2+^ levels in BC cells. In addition, flow cytometry analysis demonstrated that intracellular ROS and lipid peroxide levels in BCHE-treated BC cells were significantly increased (Fig. 3D and E). Furthermore, laser confocal microscopy revealed a remarkable increase in intracellular lipid peroxide levels in the two BC cell lines following BCHE stimulation (Fig. 3F). Taken together, BCHE induced intracellular iron and lipid peroxidation accumulation in human BC cells, leading to ferroptosis.

### BCHE stimulation inhibited glutathione peroxidase 4 (GPX4) expression and upregulated transferrin levels in human BC cells

**Figure 4:**
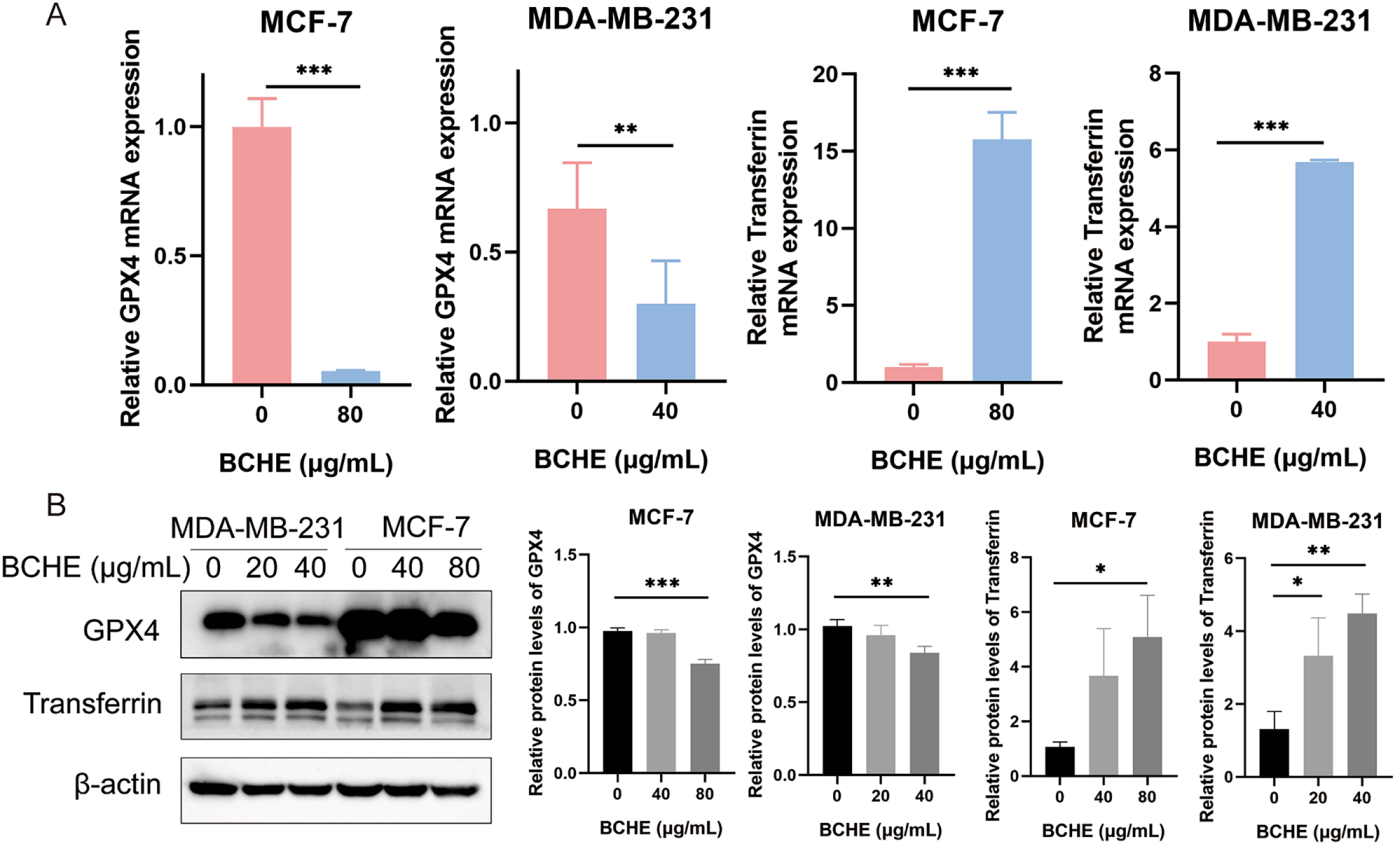
BCHE treatment inhibited GPX4 expression and upregulated Transferrin expression in human BC cells. (**a**) GPX4 and Transferrin mRNA expression in human BC cells was measured using qRT‒PCR. (**b**) GPX4 and Transferrin protein expression in human BC cells detected through Western blotting. ^*^P < 0.05, ^**^P < 0.01, ^***^P < 0.001.

To investigate the mechanism through which BCHE induces ferroptosis in BC cells, transcriptome sequencing was conducted on human BC cells treated with or without BCHE. GPX4 plays a role in the critical defense mechanism of cells that utilize glutathione (GSH) to reduce lipid peroxides to nontoxic lipid alcohols, thus exerting protective effects against ferroptosis^15^. Transferrin, also known as iron-binding protein or iron transport protein, directly participates in the transport and metabolism of iron^16^. As shown in Table 1, transcriptome sequencing analysis revealed that the abundance of GPX4 was decreased, while the abundance of Transferrin increased in BC cells after BCHE treatment. Moreover, qRT-PCR analysis was used to determine GPX4 and Transferrin expression. BCHE significantly downregulated GPX4 mRNA levels and upregulated Transferrin mRNA expression in both BC cell lines (Fig. 4A). Additionally, western blotting assays suggested that BCHE treatment decreased GPX4 protein levels and increased Transferrin protein expression in both BC cell lines (Fig. 4B). Overall, BCHE treatment decreased GPX4 levels and increased Transferrin levels in human BC cells.

**Table 1:** The expression abundance of GPX4 and Transferrin in MDA-MB-231 cells treated with or without BCHE.

Gene	Control group	BCHE group	Description
GPX4	8555	6003	Down
Transferrin	1254	2340	Up

### BCHE inhibited tumor development in a breast cancer orthotopic transplantation model

**Figure 5:**
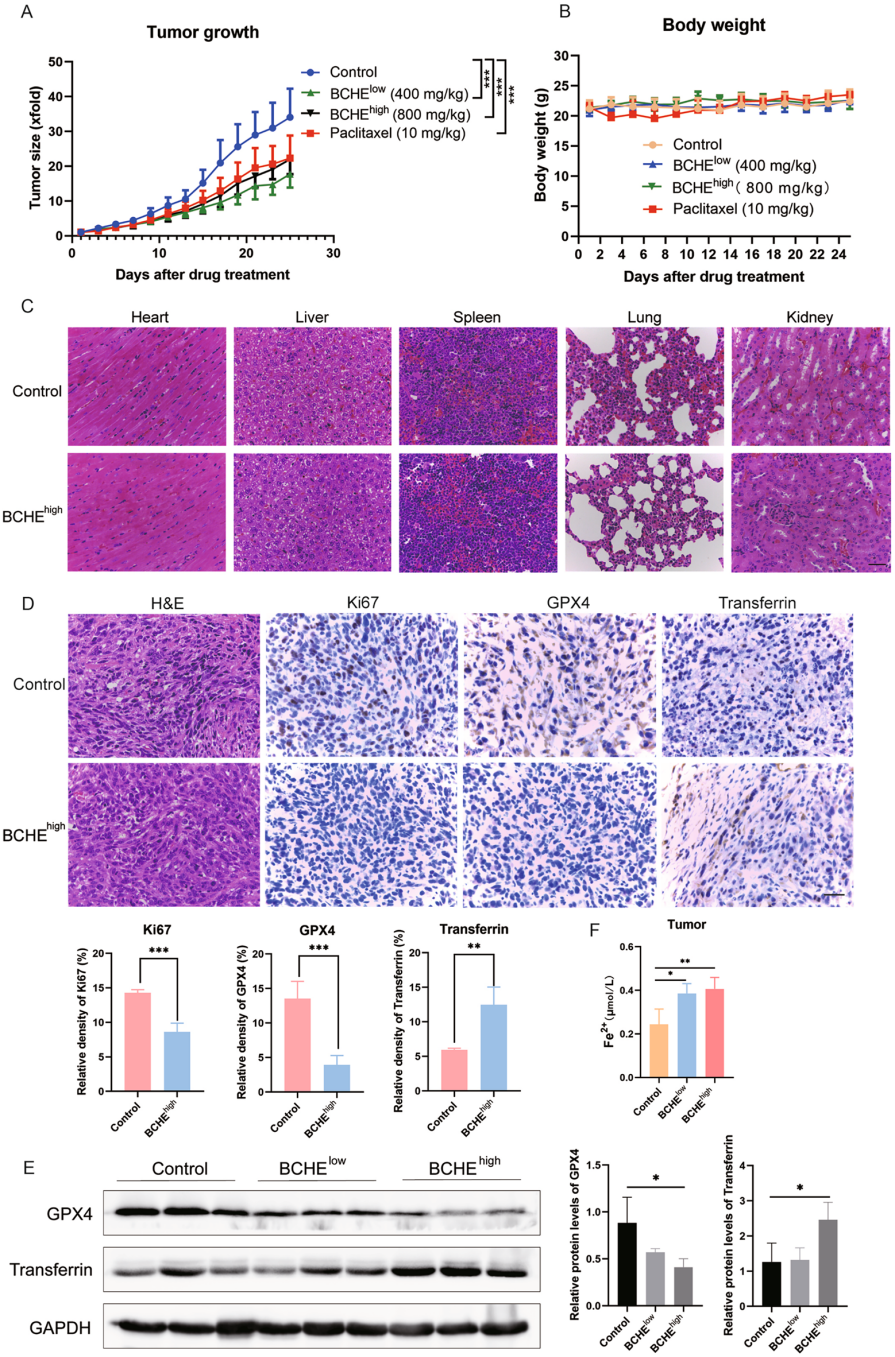
BCHE inhibited tumor development in a breast cancer orthotopic transplantation model. (**a**) Tumor volume and (**b**) body weight curves in mice subjected to treatment with PBS (oral gavage, once daily, n = 6), a low dose of BCHE (400 mg/kg, oral gavage, once daily, n = 6), a high dose of BCHE (800 mg/kg, oral gavage, once daily, n = 6), or paclitaxel (10 mg/kg, intraperitoneal injection, twice weekly, n = 6). (**c**) H&E staining of tissues from the major organs of BALB/c mice. The scale bar represents 50 µm. (**d**) IHC staining of tumor samples collected from the control and high-dose BCHE-treated groups. Scale bar = 50 µm. (**e**) Immunoblot analysis of GPX4 and transferrin expression in tumor tissues from the control, low-dose, and high-dose BCHE-treated groups. (**f**) Fe^2+^ content within tumor samples from the control, low-dose, and high-dose BCHE-treated groups. ^*^P < 0.05, ^**^P < 0.01, ^***^P < 0.001.

To evaluate the antitumor activity of BCHE in vivo, a breast cancer orthotopic transplantation model with mouse BC 4T1 cells was constructed. As shown in Fig. 5A, the administration of BCHE markedly suppressed tumor growth in the mouse orthotopic BC model. The tumor inhibition rates were approximately 47.98% and 35.54% for the high- and low-dose groups, respectively. Moreover, relative to those in the control group, the body weights of the BALB/c mice in the BCHE administration groups (Fig. 5B). H&E staining revealed no apparent injury to major organs, including the liver, heart, lung, kidney, or spleen, after BCHE treatment (Fig. 5C). Based on the results of IHC staining, the levels of the cell proliferation markers Ki67 and GPX4 were reduced in BALB/c mouse-derived tumor tissues following BCHE treatment, while transferrin expression was upregulated (Fig. 5D). Furthermore, as revealed by Western blotting, changes in GPX4 and Transferrin protein expression within tumor tissues in BALB/c mice treated with or without BCHE exhibited results consistent with those observed in in vitro experiments, wherein BCHE downregulated GPX4 expression and upregulated Transferrin expression (Fig. 5E). In addition, as shown in Fig. 5F, BCHE treatment significantly increased the Fe^2+^ content in the tumor tissues of the mice relative to that in the control group.

## Discussion

**Figure 6:**
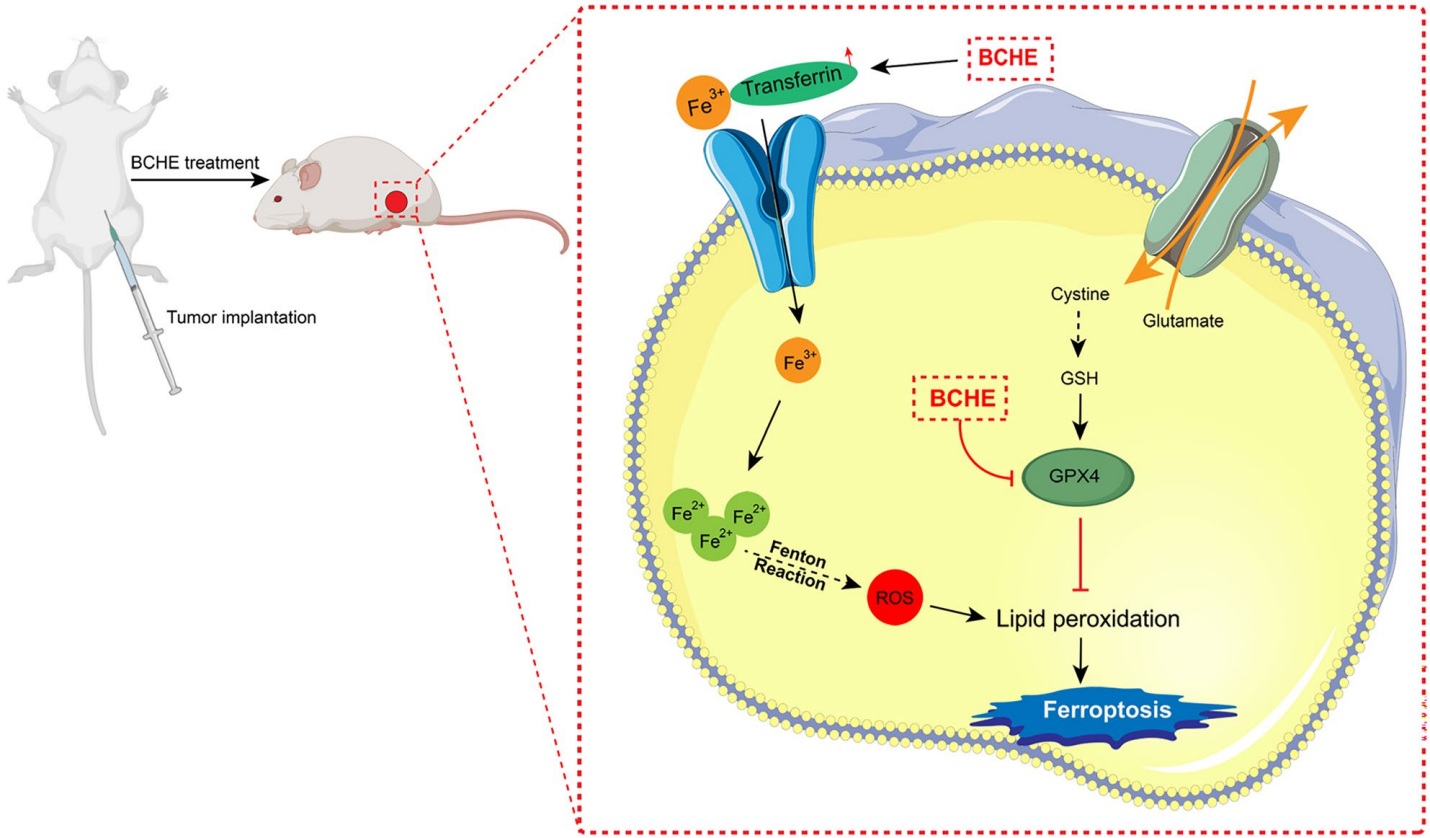
Schematic diagram illustrating mechanism of BCHE in inducing ferroptosis to exert its anti-BC effect. BCHE promotes iron transport in BC cells by increasing transferrin expression, and iron accumulation triggers the Fenton reaction, causing the generation of ROS, which serve as key mediators of intracellular lipid peroxidation. Additionally, BCHE suppresses GPX4 expression in BC cells, leading to the upregulation of ROS-induced lipid peroxidation, ultimately triggering ferroptosis in BC cells.

Currently, there is an urgent need for new therapeutic drugs to treat BC. *Boswellia carterii* has been extensively used in China to treat BC. Nevertheless, the effect of *Boswellia carterii* on treating BC remains largely unclear. There are no reports on the regulation of ferroptosis in BC cells by *Boswellia carterii* at present. This study demonstrated that BCHE exerted potent anti-BC effects via the induction of ferroptosis. BCHE upregulated Transferrin expression, leading to increased iron transport from the extracellular environment into cells, thereby increasing the iron content in BC cells. Moreover, iron accumulation within cells triggers the Fenton reaction, causing the generation of ROS, which serve as key mediators of intracellular lipid peroxidation. Additionally, BCHE suppressed GPX4 expression in BC cells, leading to the upregulation of ROS-induced lipid peroxidation (Fig. 6).

Ferroptosis is a well-known type of programmed cell death activated by ROS-induced lipid peroxidation. It is an iron-dependent process wherein the accumulation of iron in cells generates excessive ROS through the Fenton reaction^17^. Currently, some FDA-approved anticancer drugs are known to activate ferroptosis, and the antitumor efficacy of ferroptosis inducers has been demonstrated in various experimental cancer models, which highlights that ferroptosis may become a promising anticancer therapeutic approach^18^. Ferroptosis inducers effectively eliminate BC cells, and the use of ferroptosis inducers for treating BC has broad potential^8^. Celecoxib, lapatinib, quercetin, curcumin, and coumarin promote ferroptosis in BC cells^19,20^. The efficacy of traditional Chinese medicine is not determined by a single ingredient, but rather by the interaction of multiple components^21,22^. BCHE is an effective fraction extracted from *Boswellia carterii*, and its extraction rate is the highest among all extracts from *Boswellia carterii* at 48%. This suggests that the therapeutically active ingredients of *Boswellia carterii* are likely contained within BCHE. Compounds like celecoxib, lapatinib, quercetin, curcumin, and coumarin are all small molecules. In contrast, as a traditional Chinese medicine extract, BCHE is a multicomponent complex system. Compared to these small molecules, BCHE exerts its anti-tumor effect through multiple pathways, modes, and targets, with relatively fewer side effects. Ferrostatin-1 (Fer-1) is a synthetic antioxidant that prevents membrane lipid peroxidation^18^, and DFO is an iron chelator commonly used to reduce iron accumulation and deposition in tissues^15^. In this study, Fer-1 or DFO treatment impaired BCHE-mediated human BC cell death. Moreover, BCHE treatment induced intracellular iron and lipid peroxidation accumulation in human BC cells, and the administration of BCHE significantly increased the Fe^2+^ content in the tumor tissues of the orthotopic BC mouse model. Thus, BCHE is a potential inducer of ferroptosis in BC. Additionally, BCHE treatment dramatically inhibited tumor growth in a mouse orthotopic BC model. Taken together, BCHE is a potential ferroptosis-targeting candidate drug for BC therapy.

GPX4 has a vital impact on reducing lipid peroxides in the complex cell membrane environment^23^. GPX4 plays a central role in regulating ferroptosis, and GPX4 silencing promotes ferroptosis^24^. GPX4 inhibitors induced ferroptosis and blunted breast cancer progression^25^. Transferrin is an important carrier protein for iron ions and serves as the primary iron-binding glycoprotein and an intercellular iron transfer molecule. It has a crucial effect on transporting iron from absorption or storage sites to different tissues and cells^26^. As a key protein that mediates iron transfer in cells from the external environment to the internal environment, transferrin positively regulates ferroptosis^27^. Transcriptome sequencing, qRT-PCR, and immunoblotting revealed that BCHE suppressed GPX4 expression and upregulated Transferrin expression in human BC cells. Furthermore, IHC staining and immunoblotting analysis of BALB/c mouse-derived tumor tissues suggested that BCHE reduced GPX4 expression and increased transferrin expression in vivo*.* Collectively, BCHE induced ferroptosis in BC through modulating two key pathways, namely, the iron accumulation pathway and the lipid peroxidation pathway.

Collectively, BCHE exhibited potent anti-BC efficacy in vitro and in vivo via the induction of ferroptosis mediated by increased Transferrin expression and the intracellular accumulation of Fe^2+^, as well as decreased GPX4 expression and the upregulation of ROS-induced lipid peroxidation in BC cells. Therefore, as an inducer of ferroptosis, BCHE is a potential candidate drug for treating BC.

### Supplementary Information

Supplementary Figures.

Supplementary Information 1.

## Data Availability

Data is provided within the manuscript or supplementary information files.
