# Supplementary material for: Boswellia carterii n-hexane extract suppresses breast cancer growth via induction of ferroptosis by downregulated GPX4 and upregulated transferrin
Source: Sci Rep. 2024 Jun 21;14:14307. doi: 10.1038/s41598-024-65170-6 (PMC11192895; doi:10.1038/s41598-024-65170-6)
Supplement: Supplementary file 1 — Supplementary Figures. [file 41598_2024_65170_MOESM1_ESM.docx]

**Supplementary Figure S1. GC-MS Total Ion Chromatogram (TIC) of BCHE.** GC-MS (GC-MS QP-2010 Ultra, Shimadzu, Japan); High-purity Helium (He) purchased from Beijing Xianheyu Commerce and Trade Co., Ltd.; The METTLER XS105 electronic analytical balance, manufactured by Mettler-Toledo Instruments Co., Ltd., was used for the experiment. The methanol used, which was of mass spectrometry grade, was purchased from Thermo-Fisher in the United States. To prepare the samples, they were dissolved in methanol and then subjected to gradient dilution, resulting in a final concentration of 1 mg/mL. For injection, an aliquot with a volume of 1 μL was used. Chromatographic conditions: A quartz capillary column (Rtx-5MS, 30 m × 0.25 mm, 0.25 μm) was utilized. The carrier gas employed was high-purity helium (with a mass fraction of ≥ 99.999%). The temperature program was as follows: the initial temperature was set at 80°C and held for 3 minutes, followed by a ramp of 40°C per minute up to 198°C and maintained for 10 minutes. Subsequently, the temperature was increased at a rate of 1°C per minute until it reached 200°C, held for 1 minute, further increased at a rate of 10°C per minute up to 230°C, and finally ramped at a rate of 12°C per minute to 280°C and held for 7 minutes. No split flow was employed during the process. The injection port temperature was maintained at 250°C, while the detector temperature was set to 250°C. Mass spectrometric conditions: An electron ionization (EI) source was utilized, with an ion source temperature of 200°C and an ionization voltage of 70 eV. The mass scanning range was set from 35 to 400 Da, and the scanning mode was set to full scan. The scan interval was 0.1 seconds.


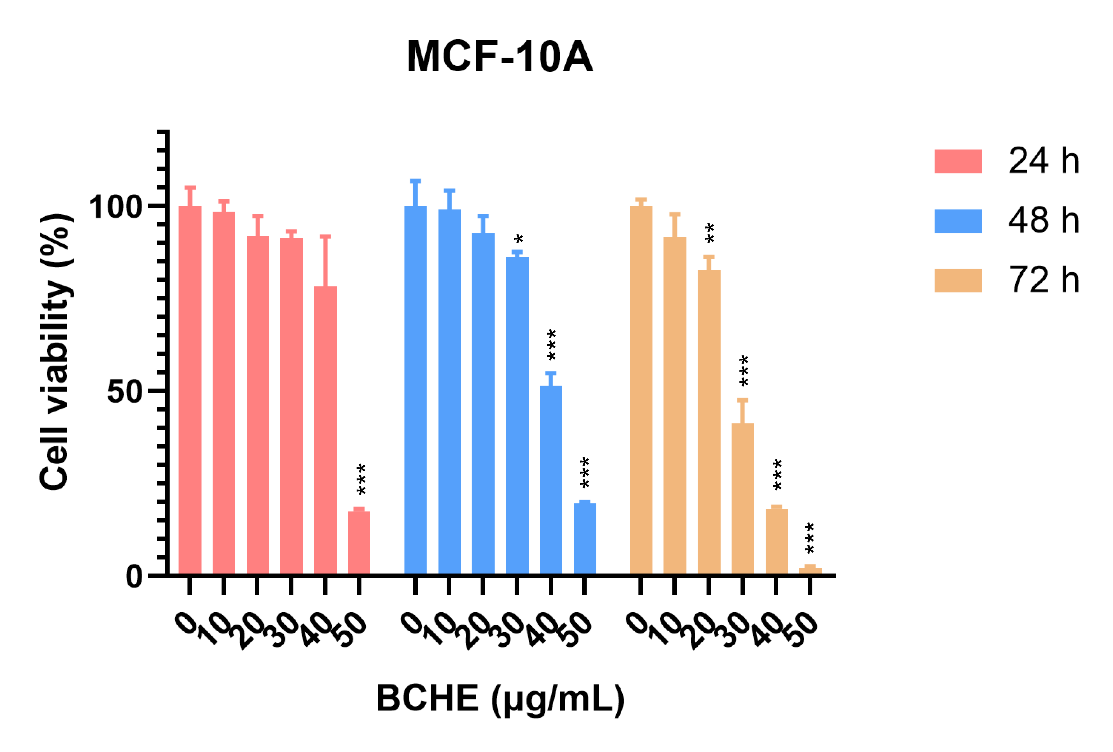


**Supplementary Figure S2. The effect of BCHE on the cell viability of human normal breast epithelial MCF-10A cells.** BCHE was added to MCF-10A cells for 24, 48, and 72 h, and a MTT assay was then conducted to analyze cell viability.
