# Supplementary material for: Boswellia carterii n-hexane extract suppresses breast cancer growth via induction of ferroptosis by downregulated GPX4 and upregulated transferrin
Source: Sci Rep. 2024 Jun 21;14:14307. doi: 10.1038/s41598-024-65170-6 (PMC11192895; doi:10.1038/s41598-024-65170-6)

**Uncropped images of western blots**

# Figure 4B

MDA-MB-231

MCF-7

BCHE(µg/ml) 0 2 4 0 2 4

**Transferrin**
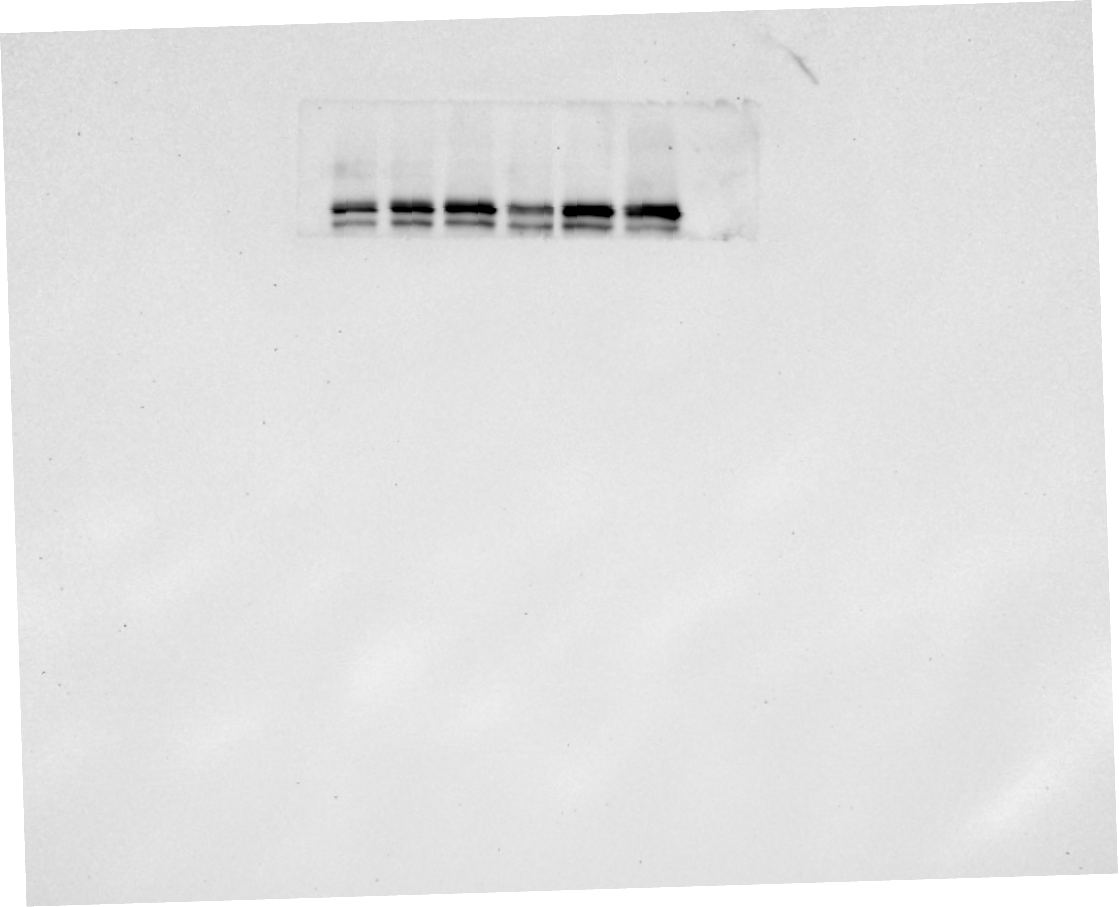


MDA-MB-231

MCF-7

BCHE(µg/ml) 0 2 4 0 2 4

**GPX4**
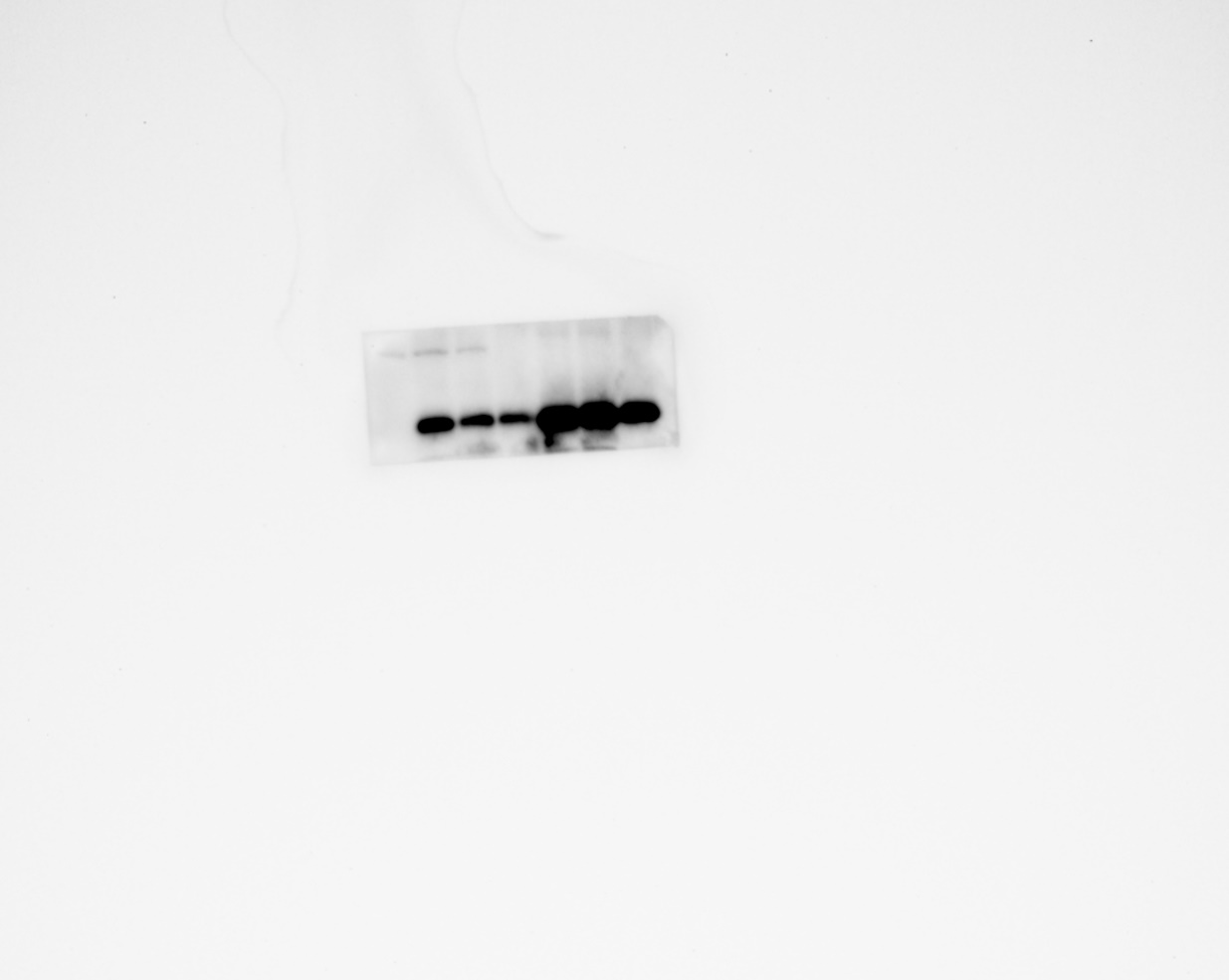


MDA-MB-231

MCF-7

BCHE(µg/ml) 0 2 4 0 2 4

**β-actin**
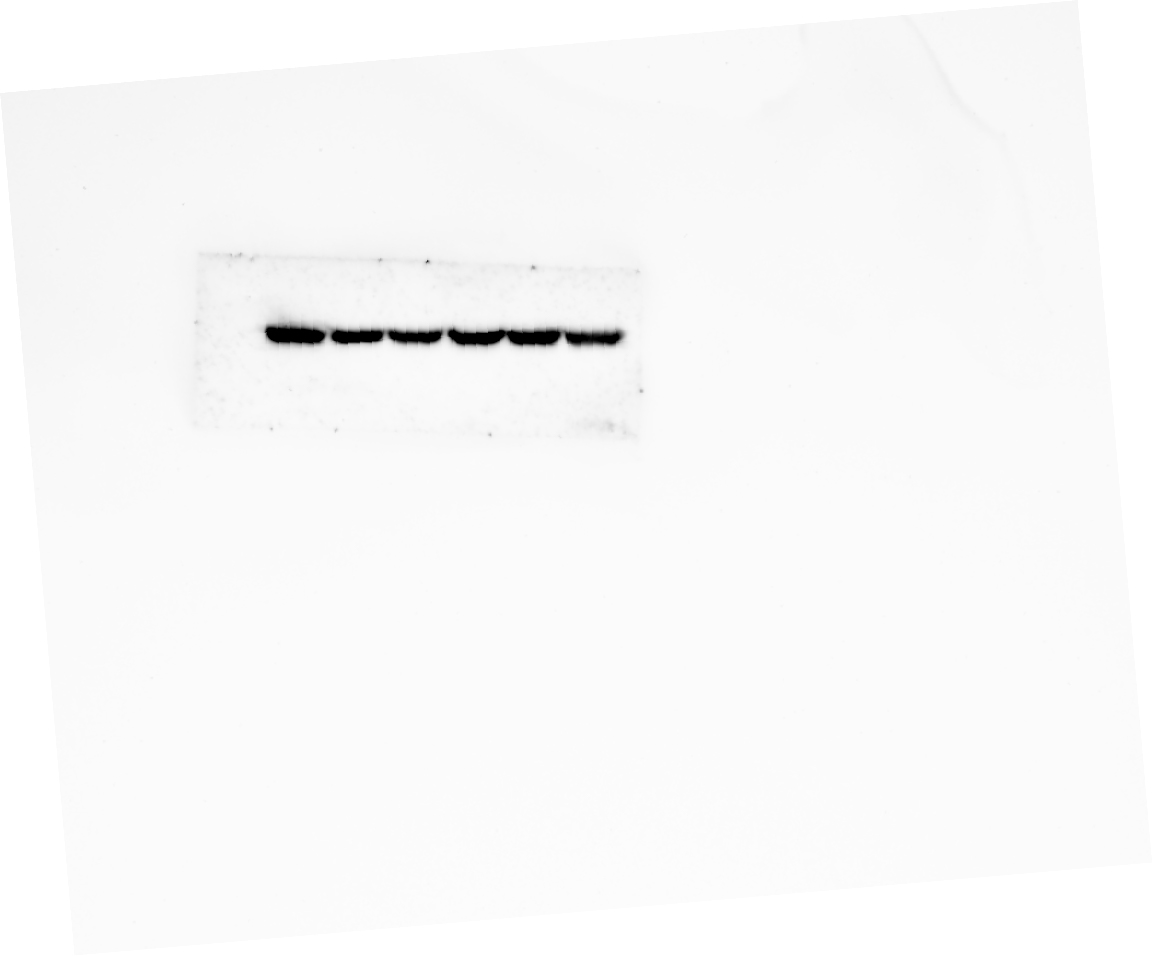


# Figure 5E

high

low

Control

BCHE

BCHE

**Transferrin**
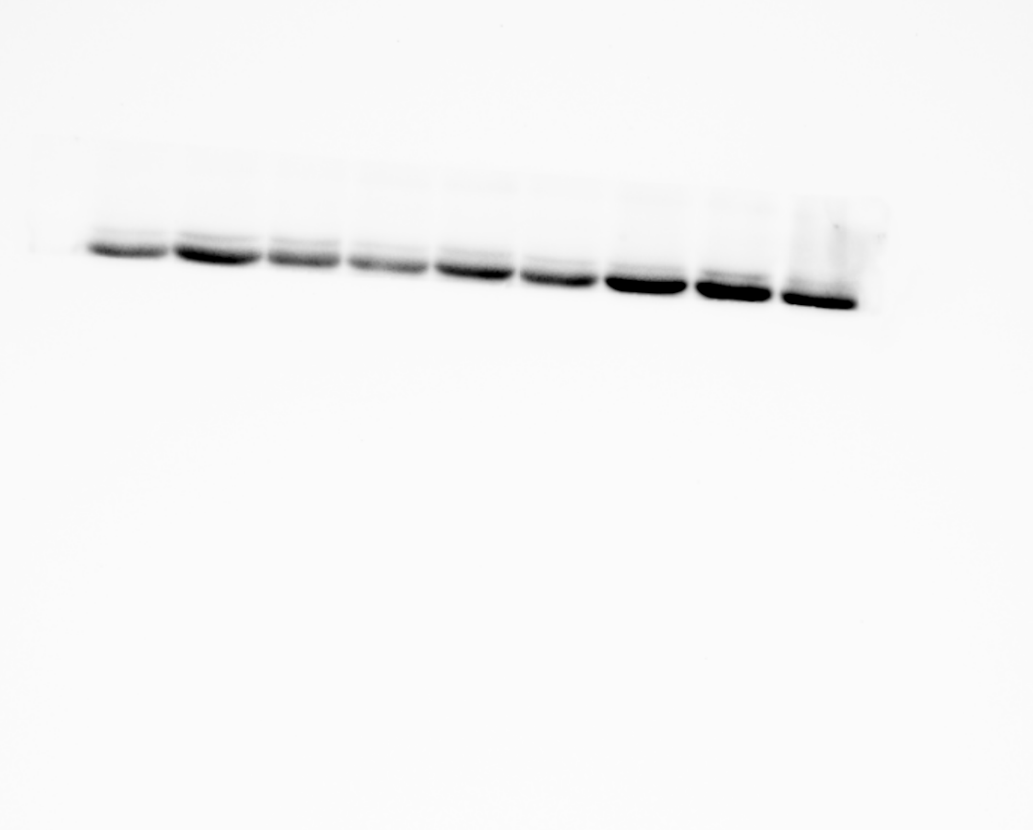


high

low

Control

BCHE

BCHE

**GPX4**
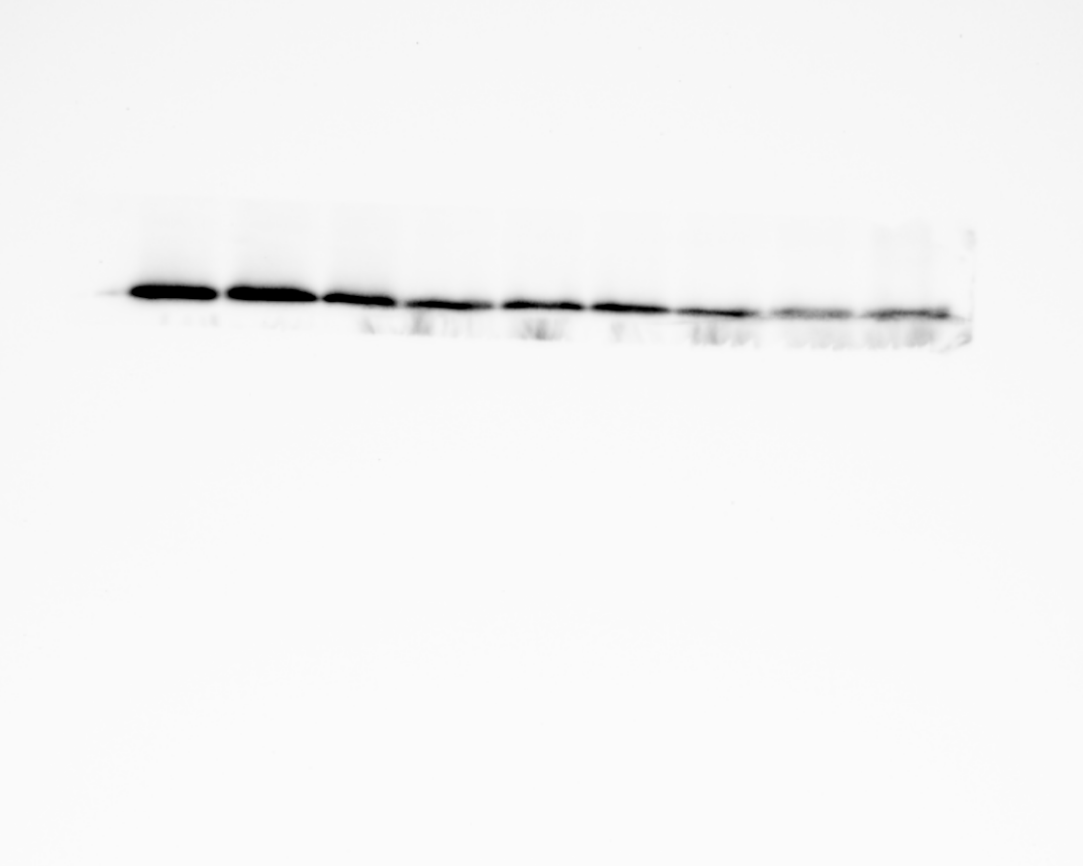


high

low

Control

BCHE

BCHE

**GAPDH**
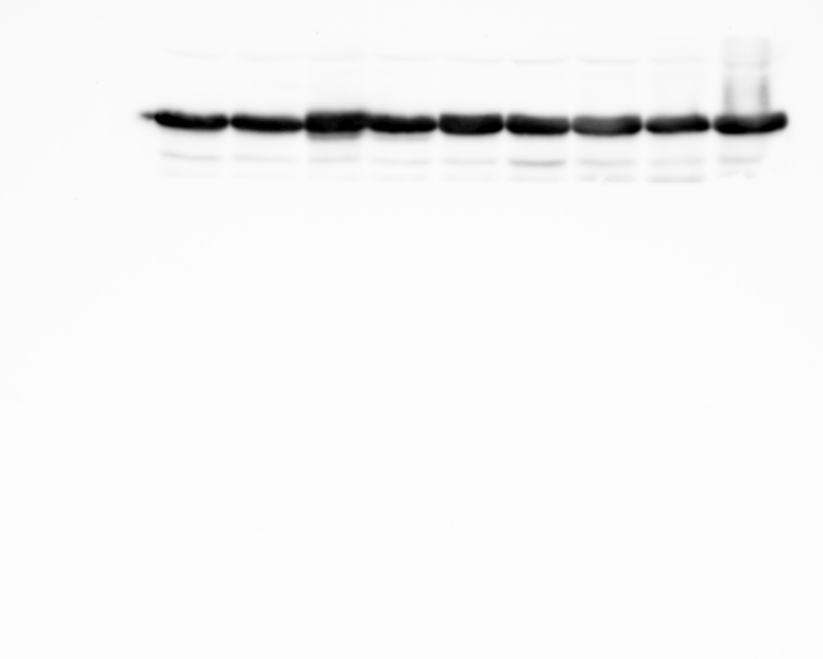

Supplement: Supplementary file 2 — Supplementary Information 1. [file 41598_2024_65170_MOESM2_ESM.docx]
